# International evidence-based guidelines on Point of Care Ultrasound (POCUS) for critically ill neonates and children issued by the POCUS Working Group of the European Society of Paediatric and Neonatal Intensive Care (ESPNIC)

**DOI:** 10.1186/s13054-020-2787-9

**Published:** 2020-02-24

**Authors:** Yogen Singh, Cecile Tissot, María V. Fraga, Nadya Yousef, Rafael Gonzalez Cortes, Jorge Lopez, Joan Sanchez-de-Toledo, Joe Brierley, Juan Mayordomo Colunga, Dusan Raffaj, Eduardo Da Cruz, Philippe Durand, Peter Kenderessy, Hans-Joerg Lang, Akira Nishisaki, Martin C. Kneyber, Pierre Tissieres, Thomas W. Conlon, Daniele De Luca

**Affiliations:** 1 Department of Paediatrics - Neonatology and Paediatric Cardiology, Cambridge University Hospitals and University of Cambridge School of Clinical Medicine, Biomedical Campus, Hills Road, Cambridge, CB2 0QQ UK; 2 Addenbrooke’s Hospital, Box 402, Cambridge, UK; 3 Paediatric Cardiology, Centre de Pédiatrie, Clinique des Grangettes, Geneva, Switzerland; 4 Department of Paediatrics, Children’s Hospital of Philadelphia and Perelman School of Medicine, Philadelphia, USA; 5 Division of Paediatrics and Neonatal Critical Care, APHP - Paris Saclay University Hospitals, “A. Béclère” Medical centre, Paris, France; 6 Department of Paediatric Intensive Care, Gregorio Marañón General University Hospital, Madrid, Spain; 7 Department of Paediatric Cardiology, Sant Joan de Déu Hospital, Barcelona, Spain; 8 Department of Paediatric Intensive Care, Great Ormond Street Hospital, London, UK; 9 Department of Paediatric Intensive Care, Hospital Universitario Central de Asturias, Oviedo. CIBER-Enfermedades Respiratorias. Instituto de Salud Carlos III, Madrid. Instituto de Investigación Sanitaria del Principado de Asturias, Oviedo, Spain; 10 Department of Paediatric Intensive Care, Nottingham University Hospitals, Nottingham, UK; 11 Department of Paediatric and Cardiac Intensive Care, Children’s Hospital Colorado, Aurora, USA; 12 Division of Paediatric Critical Care, APHP - Paris Saclay University Hospitals, “Kremlin Bicetre” Medical Centre, Paris, France; 13 Department of Paediatric Anaesthesia and Intensive Care, Children’s Hospital Banska Bystrica, Banska Bystrica, Slovakia; 14 Department of Paediatrics, Medicins Sans Frontieres (Suisse), Geneva, Switzerland; 15 Department of Anaesthesiology and Critical Care Medicine, Children’s Hospital of Philadelphia and Perelman School of Medicine, Philadelphia, USA; 16 Department of Paediatrics, Division of Paediatric Critical Care Medicine, Beatrix Children’s Hospital Groningen, University Medical Center Groningen, Groningen, The Netherlands; 17 Physiopathology and Therapeutic Innovation Unit–INSERM Unit U999, South Paris Medical School, Paris Saclay University, Paris, France

**Keywords:** Neonate, Children, Ultrasound, Point of care ultrasound (POCUS), Paediatric intensive care unit (PICU), Neonatal intensive care unit (NICU)

## Abstract

**Background:**

Point-of-care ultrasound (POCUS) is nowadays an essential tool in critical care. Its role seems more important in neonates and children where other monitoring techniques may be unavailable. POCUS Working Group of the European Society of Paediatric and Neonatal Intensive Care (ESPNIC) aimed to provide evidence-based clinical guidelines for the use of POCUS in critically ill neonates and children.

**Methods:**

Creation of an international Euro-American panel of paediatric and neonatal intensivists expert in POCUS and systematic review of relevant literature. A literature search was performed, and the level of evidence was assessed according to a GRADE method. Recommendations were developed through discussions managed following a Quaker-based consensus technique and evaluating appropriateness using a modified blind RAND/UCLA voting method. AGREE statement was followed to prepare this document.

**Results:**

Panellists agreed on 39 out of 41 recommendations for the use of cardiac, lung, vascular, cerebral and abdominal POCUS in critically ill neonates and children. Recommendations were mostly (28 out of 39) based on moderate quality of evidence (B and C).

**Conclusions:**

Evidence-based guidelines for the use of POCUS in critically ill neonates and children are now available. They will be useful to optimise the use of POCUS, training programs and further research, which are urgently needed given the weak quality of evidence available.

## Introduction

The incorporation of Point of Care Ultrasound (POCUS) has represented a transformative change in clinical practice, challenging the traditional diagnostic and procedural “art” of medicine, especially in acute care environments. It has emerged as “the new” clinical tool and it has displaced, in some way, the classical stethoscope. It is performed and interpreted by the bedside clinician with the intent of either answering a focused question or achieving a specific procedural goal.

The use of POCUS by critical care providers has increased significantly in recent years and adult emergency medicine had pioneered this field with the publication of guidelines for implementation of structured POCUS training [1–7]. These efforts were translated into paediatrics through emergency medicine. Since then, POCUS has gained popularity and expanded across many paediatric disciplines. However, unified guidelines on the use of POCUS and training do not exist yet for the paediatric and neonatal critical care.

Bedside goal-directed echocardiography has been the first POCUS application in paediatric practice with guidelines for implementation. An expert statement was published in 2011, followed by the United Kingdom Expert Consensus Statement on Neonatologist Performed Echocardiography (NPE) and recommendations for NPE in Europe [8–10]. The Australian Clinician Performed Ultrasound (CPU) programme is a well-established academic curriculum developed to train neonatologists in the use of POCUS, though applications are limited to the evaluation of the neonatal brain and heart [10, 11] and the training programme is restricted to neonatal physiology. Non-cardiac POCUS may carry more opportunities for use as well as a number of benefits for both patients and providers. However, the lack of guidelines is a barrier to widespread adoption.

As more acute care providers invest in POCUS technology, it is critically important to define POCUS main purposes, establish formal training and develop accreditation guidelines, in order to maximise POCUS benefits and reducing risks.

Therefore, the European Society for Paediatric and Neonatal Intensive Care (ESPNIC) assembled a group of international paediatric POCUS key opinion leaders to create evidence-based guidelines for the use of current and emerging POCUS applications in the neonatal (NICU) and paediatric intensive care units (PICU) by any clinician working in these units.

## Methods

Three lead authors, one neonatologist (YS), one paediatric intensivist (DDL) and one paediatric cardiologist (CT), identified expert colleagues who significantly contributed with publications in the POCUS field and/or have developed POCUS training courses in the last 10 years, similarly to what had been done with previous ESPNIC guidelines [12]. Panellists selection was performed prior to the literature search and for logistic reasons, the number of participants was limited to a maximum of 20. These colleagues should have been fairly representative of all POCUS fields and both Europe and North America and include also non-ESPNIC members. Moreover, at least one co-author should have been an expert in guidelines development and supervised the whole methodology, while literature search was performed by each panellist for their sub-section. All invited experts agreed to participate. Details of the methods used to produce these guidelines are given in the additional file for online supplementary material (see Additional file 1). These guidelines followed relevant ESPNIC internal procedures for manuscript endorsement, and this included an external review by ESPNIC officers not included among the panellists. Guidelines have been prepared according to the international Appraisal of Guidelines, Research and Evaluation (AGREE) [13]. Each recommendation is intended to be applied both for paediatric and neonatal patients, unless otherwise specified.

## Results

**Fig. 1:**
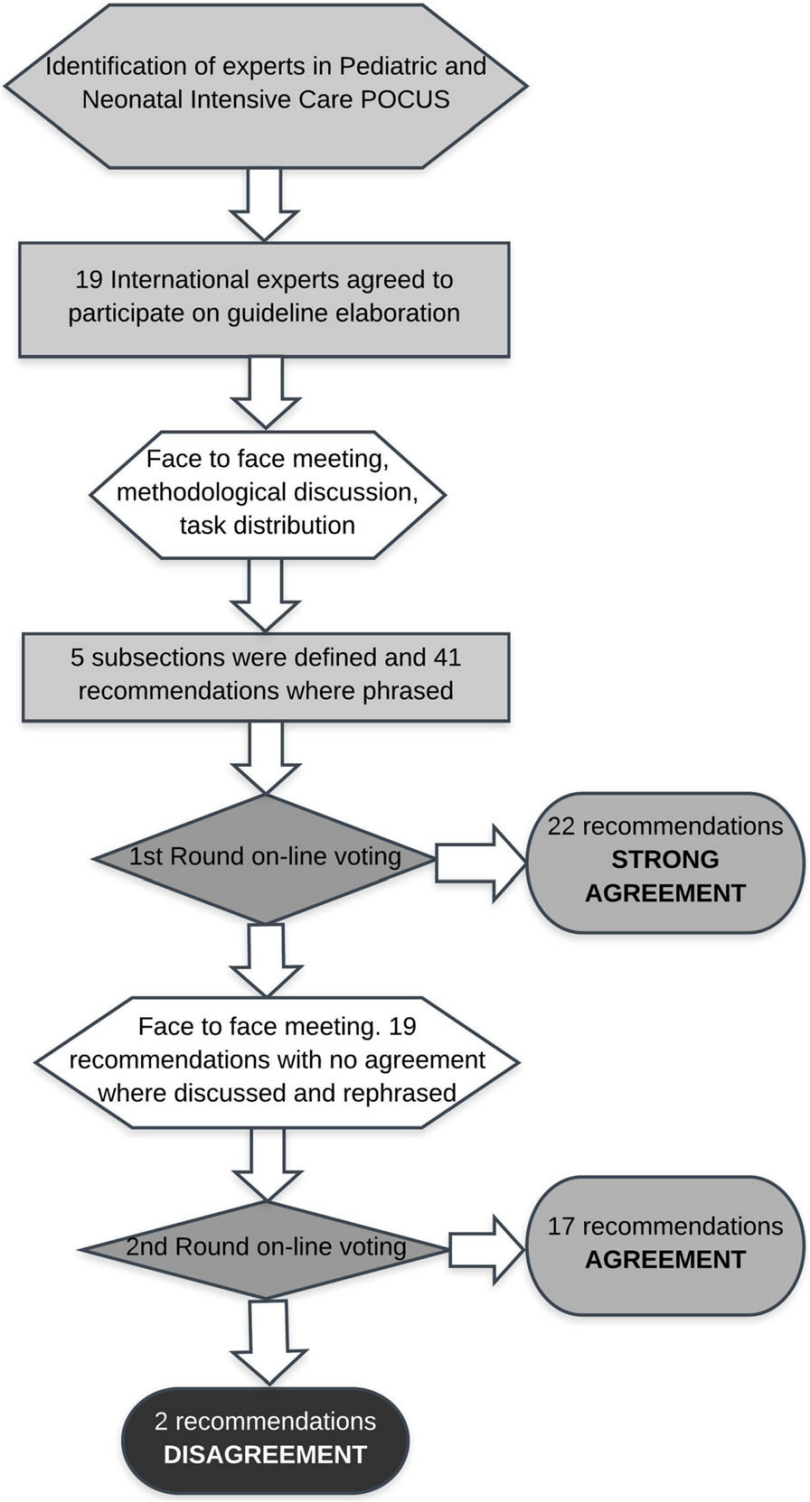
Flow chart of the methodology used in POCUS guidelines and consensus development

A total of 41 recommendations on the use of POCUS in neonatal and paediatric critical care were discussed. There was strong agreement on 22 recommendations after first-round voting. Following the discussion in an in-person meeting among the experts (organised within the 2018 ESPNIC congress (Paris, France)), 19 recommendations were re-worded and re-voted in the second round (Fig. 1). There was agreement on 17 recommendations and disagreement on 2 recommendations as summarised in Table 1 and discussed below.

A. Recommendations for use of cardiac POCUS
  1. POCUS should not be used as a screening tool for diagnosing congenital heart defects in neonates and children, unless neonatologists/paediatric intensivists have received an advanced echocardiography training specifically for this purpose—*strong agreement* (*quality of evidence A*). The goal of cardiac POCUS is to obtain physiological and haemodynamic information to aid in clinical decision making. If any structural abnormality are suspected or diagnosed while performing cardiac POCUS, a formal echocardiogram should be performed by a paediatric cardiologist [8–11, 14]. POCUS should not be used as a screening tool for diagnosing congenital heart defects, unless neonatologists/paediatric intensivists have received an advanced echocardiography training specifically for this purpose.
  2. POCUS may be helpful to assess cardiac filling (preload assessment) and intravascular volume status in neonates and children—*strong agreement (quality of evidence D)*. Cardiac filling may be qualitatively assessed in apical windows and semi-quantitatively assessed by interrogating inferior vena-cava (IVC) size and variation during the cardiorespiratory cycle. During this evaluation, the eventual presence of right heart failure or elevated intra-abdominal pressure should be taken into consideration. In non-ventilated adults and children with normal right atrial pressure, IVC collapse during inspiration is > 50%. A dilated IVC with decreased collapsibility (< 50%) is a sign of increased right atrial pressure (above 10 mmHg) [15–17]. Conversely, a collapsed IVC may be suggestive of hypovolemia. Clinicians performing POCUS should be aware of its limitations especially in children on mechanical ventilation and neonates (especially when an umbilical central venous catheter is placed) in whom no validated normative data yet exist [18, 19]. Moreover, mechanical ventilation (especially with high mean airway pressure), spontaneous breathing effort and some concomitant conditions may reduce the reliability of IVC evaluation to predict fluid responsiveness [20].
  3. POCUS may be helpful to assess fluid responsiveness in neonates and children—*strong agreement (quality of evidence D)*. The variation of velocity-time integrals (VTIs) measured at the left ventricular outflow tract (LVOT), using pulse wave Doppler (PWD) in apical 5-chamber view, during inspiration and expiration has been reported to predict volume responsiveness. A variation of > 15% has been reported to have a high predictive value with a sensitivity and specificity exceeding 90% [21, 22]. This has been validated in several studies, including many studies involving mechanically ventilated children [16, 23–31]. Accurate measurement of VTI needs acquisition of apical 5-chamber view and good alignment of ultrasound beam with the across LVOT like any other Doppler assessment, which requires advanced ultrasonography skills. Hence this recommendation is particularly for neonatal/paediatric intensivists with advanced POCUS skills.
  4. POCUS may be helpful for qualitative assessment of cardiac function on visual inspection in neonates and children—*strong agreement (quality of evidence D)*. POCUS should be used for qualitative assessment of cardiac contractility (“eyeballing” assessment) in multiple views such as apical 4-chamber (A4C), parasternal long (PLAX) and short-axis (PSAX) and sub-costal short-axis views [8, 15]. Sub-costal views may be very helpful in patients where parasternal or apical views impossible or sub-optimal, and in younger children or neonates where sub-costal views may provide even better image quality.
  5. POCUS is helpful for semi-quantitative assessment of cardiac function in neonates and children [however, a detailed functional assessment should be performed by a person with advanced echocardiography training]—*agreement (quality of evidence C)*. POCUS can be used to assess cardiac function semi-quantitatively by assessing ejection fraction (EF) and fraction shortening (FS) [14, 15]. In experienced hands, POCUS can be used for further assessment of left and right ventricular function as summarised in Table 2 [14, 15, 32–45]. However, a detailed assessment of cardiac function should be performed by a neonatologist/paediatric intensivist with advanced echocardiography training or a paediatric cardiologist.
  6. POCUS is helpful for the assessment of pulmonary artery systolic pressure in pulmonary hypertension in neonates and children—*strong agreement (quality of evidence B)*. In the presence of tricuspid regurgitation, pulmonary artery systolic pressure (PASP) can be reliably estimated using bedside POCUS. In the absence of right outflow tract obstruction, the regurgitation velocity represents the difference in pressure between the right atrium and the right ventricle. PASP is derived using a modified Bernoulli equation [14, 15, 32].
  7. POCUS is helpful for semi-quantitative assessment of pulmonary hypertension in neonates and children—*strong agreement (quality of evidence B).* In the absence of tricuspid regurgitation, POCUS can be used for semi-quantitative assessment of pulmonary hypertension by evaluating the interventricular septal position and movement at the end of systole and by assessing flow direction and velocities across a patent *ductus arteriosus* and/or *foramen ovale* [14, 15].
  8. POCUS is helpful to diagnose pericardial effusion in neonates and children—*strong agreement (quality of evidence B)*. Pericardial effusion can reliably be seen on POCUS by using multiple echocardiographic views [15, 46]. Cardiac tamponade should be detected within the SAFE (Sonographic Assessment of liFe threatening Events) algorithm dedicated to recognise the main causes of life-threatening events in children [47].
  9. POCUS is helpful to guide pericardiocentesis in neonates and children—*strong agreement (quality of evidence B)*. Ultrasound-guided pericardiocentesis has lower complication rates compared to the traditional landmark technique [48–50].
  10. POCUS should be used to assess the patency of *ductus arteriosus*—*strong agreement (quality of evidence A)*. Cardiac POCUS can reliably be used to assess patency of *ductus arteriosus* in preterm infants [8–10]. In experienced hands, it may be used for hemodynamic evaluation of the *ductus arteriosus* [8, 10].
  11. POCUS may be used to detect vegetations to make a diagnosis of endocarditis [however, a definitive diagnosis requires a detailed assessment by a paediatric cardiologist or a person with advanced echocardiography training]*—disagreement (quality of evidence D).* There was no agreement on using POCUS for the diagnosis of endocarditis. Detecting vegetations to make a diagnosis of endocarditis should be done by more expert (paediatric cardiologist or by an intensivist with advanced POCUS skills, and not expected to rule out by neonatal and paediatric intensivists with competencies in basic POCUS). However, intracardiac masses may be detected on POCUS examination and should be referred to a paediatric cardiologist or a neonatologist/paediatric intensivist with advanced echocardiography training [51, 52].

**Table 1:** Summary of recommendations on the use of POCUS in the neonatal and paediatric critical care

No.	Recommendation	Level of agreement	Quality of evidence
1.	POCUS should not be used as a screening tool for diagnosing congenital heart defects in neonates and children, unless neonatologists/paediatric intensivists have received an advanced echocardiography training specifically for this purpose	Strong agreement	A
2.	POCUS may be helpful to assess cardiac filling (preload assessment) and intravascular volume status in neonates and children	Strong agreement	D
3.	POCUS may be helpful to assess fluid responsiveness in neonates and children	Strong agreement	D
4.	POCUS may be helpful for qualitative assessment of cardiac function on visual inspection in neonates and children	Strong agreement	D
5.	POCUS is helpful for semi-quantitative assessment of cardiac function in neonates and children [however, a detailed functional assessment should be performed by a person with advanced echocardiography training]	Agreement	C
6.	POCUS is helpful for assessment of pulmonary artery systolic pressure in pulmonary hypertension in neonates and children	Strong agreement	B
7.	POCUS is helpful for semi-quantitative assessment of pulmonary hypertension in neonates and children	Strong agreement	B
8.	POCUS is helpful to diagnose pericardial effusion in neonates and children	Strong agreement	B
9.	POCUS is helpful to guide pericardiocentesis in neonates and children	Strong agreement	B
10.	POCUS should be used to assess the patency of *ductus arteriosus* in neonates and children	Strong agreement	A
11.	POCUS may be used to detect vegetation to make or exclude the diagnosis of endocarditis [however, a definitive diagnosis requires a detailed assessment by a paediatric cardiologist].	Disagreement	D
12.	POCUS is helpful to distinguish between respiratory distress syndrome (RDS) and transient tachypnoea of the neonate (TTN)	Agreement	B
13.	POCUS is helpful to detect pneumonia in neonates and children	Agreement	B
14.	POCUS is helpful to semi-quantitatively evaluate lung aeration and help the management of respiratory intervention in acute respiratory distress syndrome (ARDS) in neonates and children	Agreement	B
15.	POCUS is helpful to recognise meconium aspiration syndrome (MAS)	Agreement	C
16.	POCUS is helpful for descriptive purposes in viral bronchiolitis but cannot provide a differential aetiological diagnosis	Strong agreement	C
17.	POCUS is helpful to accurately detect pneumothorax in neonates and children	Strong agreement	B
18.	POCUS is helpful to insert chest tube or perform needle aspiration in neonatal tension pneumothorax	Strong agreement	B
19.	POCUS is helpful to detect pleural effusions in neonates and children	Strong agreement	B
20.	POCUS is helpful to guide thoracentesis in neonates and children	Strong agreement	B
21.	POCUS is helpful to evaluate lung oedema in neonates and children	Agreement	C
22.	POCUS is helpful in detecting anaesthesia-induced atelectasis in neonates and children	Agreement	C
23.	POCUS-guided technique should be used for internal jugular vein (IJV) line placement in neonates and children	Strong agreement	A
24.	POCUS-guided technique is helpful for subclavian vein line placement in neonates and children	Strong agreement	B
25.	POCUS-guided technique is helpful for femoral line placement in neonates and children	Strong agreement	B
26.	POCUS-guided technique is helpful for arterial catheters placement in children	Agreement	B
27.	POCUS-guided technique is helpful for peripherally inserted central catheters in children	Agreement	B
28.	POCUS is helpful to locate catheter tip position in neonates and children	Strong agreement	C
29.	POCUS is helpful to detect cerebral blood flow changes in neonates and children	Agreement	B
30.	POCUS should be used to detect germinal matrix and intraventricular haemorrhage (IVH) in neonates	Strong agreement	A
31.	POCUS is helpful to detect cerebral blood flow patterns suggesting the presence of cerebral circulatory arrest in children with fused skull bones	Agreement	C
32.	POCUS is helpful to detect cerebral blood flow changes secondary to vasospasm in patients with traumatic brain injury and non-traumatic intracranial bleeding.	Agreement	C
33.	POCUS is helpful to detect changes in optic nerve sheath diameter (ONSD) indicative of raised ICP in children with fused skull bones	Agreement	B
34.	POCUS is helpful to detect cerebral midline shift in neonates and children	Agreement	C
35.	POCUS is helpful for detection of free intra-abdominal fluid in neonates and children	Strong agreement	C
36.	POCUS may detect parenchymal changes of abdominal organs in neonates and children [although for a definitive diagnosis a detailed assessment should be performed by a paediatric radiologist]	Agreement	D
37.	POCUS may detect obstructive uropathy in neonates and children [although for a definitive diagnosis a detailed assessment should be performed by a paediatric radiologist]	Agreement	D
38.	POCUS may assess bowel peristalsis in neonates and children	Agreement	D
39.	POCUS may recognise hypertrophic pyloric stenosis [although for a definitive diagnosis a detailed assessment should be performed by a paediatric radiologist]	Disagreement	D
40.	POCUS my guide peritoneal drainage or aspiration of peritoneal fluid in neonates and children	Strong agreement	D
41.	POCUS is helpful to detect signs of necrotising enterocolitis [although for a definitive diagnosis a detailed assessment should be performed by a paediatric radiologist or a person with specific advanced ultrasound training]	Agreement	C

**Table 2:** Semi-quantitative systolic ventricular function measures that might be used by the clinician with more evolved training in cardiac POCUS. Normative values are taken from the available literature on the topic [32–45] and represent the best reference data available so far, although, in some cases, specific level for a different class of patients’ age are lacking

Parameter	View	Measurement	Reference values
LV fraction shortening (FS%)	PSAX, PLAX, (2D or M mode)	LV intraluminal diameter change	28–46% for all ages
LV ejection fraction (Simpson’s method)	A4C, A2C	Percentage change of LV volume between end-diastole and end-systole	55–80% for all ages
E-point septal separation (EPSS)	PLAX (2D or M mode)	Distance between anterior leaflet of the mitral valve and intraventricular septum during the diastolic phase. This measurement is associated with LV systolic volume.	> 7 mm in adults predictive of severe LV dysfunction *
LV output (stroke volume)	A5C, PLAX	Product of VTI measured by pulse wave Doppler at LVOT in A5C and LVOT cross-sectional area measured in PLAX	*Z*-scores available for different ages and should be used; neonates: 150–400 ml/kg/min
Mitral annular plane systolic excursion (MAPSE)	A4C	Systolic excursion of lateral (or medial) mitral annulus toward apex to assess LV systolic function.	*Z*-scores available for different ages and should be used; term neonates: > 8 mm (8–11 mm) Adults 12–14 mm (< 8 mm predictive of severe LV dysfunction)
RV output (stroke volume)	PSAX or sweep PLAX	Product of VTI measured by pulsed-wave Doppler at RVOT and RVOT cross-sectional area	*Z*-scores available for different ages and should be used; neonates: 150–400 ml/kg/min
Tricuspid annular plane systolic excursion (TAPSE)	A4C	Systolic excursion of lateral (or medial) tricuspid annulus toward apex to assess RV systolic function.	Term neonates: > 8 mm (8–11 mm) Children–*Z*-score available; generally > 12 mm (12–17 mm) Adults or grown-up children > 17 mm (17–25 mm)

*A4C* Apical 4 chamber view, *A5C* Apical 5 chamber view, A2C Apical 2 chamber view, *PSAX* parasternal short-axis view, *PLAX* parasternal long-axis view, *M mode* motion mode, *LV* left ventricle, *LVOT* left ventricular outflow tract, *VTI* velocity time integral

*No data are available in neonates or children

Semi-quantitative systolic ventricular function measures that may be used in POCUS have been summarised in Table 2 [32–45].

B. Recommendations for use of lung POCUS
  1. POCUS is helpful to distinguish between respiratory distress syndrome (RDS) and transient tachypnoea of the neonate (TTN)—*agreement (quality of evidence B).* RDS is characterised by a poorly aerated lung with the absence of A-lines, presence of small “subpleural” consolidations and diffuse white lung (confluent B-lines). Conversely, in TTN, the interstitial pattern alternates with areas of near-normal lung (with A-lines). Pleural line thickening might be seen in late preterm and term babies [53–61]. The double lung point has been proposed as a pathognomonic finding [62, 63] but it is debated, as it does not seem necessary for TTN diagnosis if normal lung areas are evident [64]. High inter-observer agreement between physicians with different lung ultrasound (LUS) expertise has been reported, which makes the differential diagnosis between RDS and TTN reliable, irrespective of the operator [65]. In preterm neonates with RDS, various studies showed that a semiquantitative ultrasound evaluation of lung aeration is very predictive of the need for surfactant [66, 67] and, therefore, this POCUS tool is helpful to decide about surfactant replacement and improve its timeliness [68].
  2. POCUS is helpful to detect pneumonia in neonates and children—*agreement (quality of evidence B).* LUS signs of pneumonia are the presence of consolidations and dynamic air bronchograms, B-lines and pleural effusion. Abnormal pleural line and decreased lung sliding may be observed [58, 65]. LUS has been reported to have higher diagnostic accuracy compared with chest X-rays for the diagnosis of pneumonia [69]. However, there is no defined threshold for consolidation size or a consensual method of measurement.
  3. POCUS is helpful to semi-quantitatively evaluate lung aeration and help the management of respiratory intervention in acute respiratory distress syndrome (ARDS) in neonates and children—*agreement (quality of evidence B).* LUS in neonatal and paediatric ARDS shows bilateral diffuse areas of reduced lung aeration with areas of the interstitial syndrome and consolidations, pleural line abnormalities and pleural effusion [70–73]. Although current diagnostic criteria for ARDS do not yet include LUS, it represents a useful tool for its detection [73, 74]. Several LUS aeration scores are used to semi-quantitatively measure the effect of fluid restriction, alveolar recruitment and surfactant administration [75–79]. Scores based on the main lung ultrasound semiology (including A lines, alveolar-interstitial pattern and presence of consolidations) should be preferred over the simple B lines count, as they describe better the lung aeration and have been validated with various techniques [80].
  4. POCUS is helpful to recognise meconium aspiration syndrome (MAS)—*agreement (quality of evidence C).* MAS is now recognised as a cause of neonatal ARDS [73] and shares the same LUS findings. However, this LUS pattern is dynamic and changes with the spread of meconium plugs during mechanical ventilation [81, 82].
  5. POCUS is helpful for descriptive purposes in viral bronchiolitis but cannot provide a differential aetiological diagnosis—*strong agreement (quality of evidence C).* LUS signs in viral bronchiolitis consist of pleural line irregularities, “sub-pleural” consolidations and areas with interstitial pattern [83–85]. Good concordance between operators has been shown in wheezing infants [86]. LUS findings in viral bronchiolitis are similar to those seen during influenza outbreaks [87] but it is not currently possible to differentiate between different forms of viral respiratory infections.
  6. POCUS is helpful to accurately detect pneumothorax in neonates and children—*strong agreement (quality of evidence B).* In adults, LUS has a high diagnostic accuracy for the diagnosis of pneumothorax [88, 89] and has been reported to be more sensitive than conventional radiology [90]. Neonatal data confirm this high diagnostic performance for tension pneumothorax [91].
  7. POCUS is helpful to insert chest tube or perform needle aspiration in neonatal tension pneumothorax—*strong agreement (quality of evidence B)*. LUS should not only be used to diagnose pneumothorax but also to provide static guidance for pleurocentesis [91]. Thus, POCUS should be used to identify lung margin, hemidiaphragm and sub-diaphragmatic organs throughout the respiratory cycle before needle or tube insertion to safely avoid them.
  8. POCUS is helpful to detect pleural effusions in neonates and children—*strong agreement (quality of evidence B).* Policy statements in adults recommend the use of LUS for the detection of effusions and evaluation of pleural fluid volume to guide management [55, 92, 93]. In children, LUS shows high accuracy in the diagnosis of pneumonia-related pleural effusion [47].
  9. POCUS is helpful to guide thoracentesis in neonates and children—*strong agreement (quality of evidence B).* Ultrasound-guided thoracentesis reduces the risk of complications and increases success rates [55, 92, 93]. It should be used to identify lung margin, hemidiaphragm and sub-diaphragmatic organs throughout the respiratory cycle before needle or tube insertion to safely avoid them.
  10. POCUS is helpful to evaluate lung oedema in neonates and children—*agreement (quality of evidence C)*. Although LUS is accurate in detecting extra-vascular lung fluid [94], it cannot distinguish between cardiogenic and non-cardiogenic oedema [55, 94]. LUS has been used in children to evaluate cardiogenic lung oedema, by assessing extravascular lung fluid counting numbers of B-lines after cardiac surgery [79]. However, extravascular lung water may obviously be affected by the pressure delivered during mechanical ventilation. Thus, in these conditions, LUS globally evaluates the lung aeration rather than extravascular lung fluid.
  11. POCUS is helpful in detecting anaesthesia-induced atelectasis in neonates and children—*agreement (quality of evidence C).* LUS may be used to monitor this complication during anaesthesia that may potentially lead to hypoxemia [95].
C. Recommendations for use of vascular POCUS in line placement
  1. POCUS-guided technique should be used for internal jugular vein (IJV) line placement in neonates and children—*strong agreement* (*quality of evidence A*). Robust paediatric data favour ultrasound guidance for IJV cannulation compared to landmark technique [96–100]. Multiple studies have repeatedly shown decreased risk of cannulation failure and arterial puncture, higher success rates on first attempt and decreased incidence of complications [100–104].
  2. POCUS-guided technique is helpful for subclavian venous line placement in neonates and children—*strong agreement (quality of evidence B)*. The subclavian and brachiocephalic veins have been cannulated in children and neonates (including preterm ones) with in-plane visualisation, and this seems the easiest approach [105–107]. However, there is no firm consensus about the best technique, i.e., supra-clavicular vs infra-clavicular, or in-plane vs out-of-plane [101, 108]. Multiple cases series of children and neonates reported higher success rates and decreased incidence of complications favouring ultrasound use compared to landmark technique. Therefore, ultrasound-guided subclavian cannulation in neonates and children is safe, doable and is advised over a blind cannulation technique [101, 107–112].
  3. POCUS-guided technique is helpful for femoral line placement in neonates and children—*strong agreement* (*quality of evidence B*). Paediatric trials comparing US-guided femoral line placement with landmark technique showed higher overall success rate and on the first attempt as well as fewer needle passes [113–116].
  4. POCUS-guided technique is helpful for arterial catheters placement in children—*agreement* (*quality of evidence B*): a few high-quality studies have found that US-guided arterial line placement is faster (shorter time to success and lower number of attempts) and has higher first-attempt cannulation rates regardless of the location [117, 118].
  5. POCUS-guided technique is helpful for peripherally inserted central catheters (PICC) in children—*agreement* (*quality of evidence B*). A paediatric RCT comparing US-guided versus landmark PICC placement showed higher first-attempt cannulation rate, successful PICC positioning rate and shorter time to success when ultrasound was used [119]. Similar findings are supported by adult literature [120].
  6. POCUS is helpful to locate catheter tip position in neonates and children—*strong agreement* (*quality of evidence C*). A trial and two observational studies in the paediatric and neonatal population have suggested that US may decrease radiation and number of line manipulations by confirming PICC tip position after placement [121–123] and could be considered a complement to conventional radiography [124].
D. Recommendations for use of cerebral POCUS
  1. POCUS is helpful to detect cerebral blood flow changes in neonates and children—*agreement* (*quality of evidence B*). Children are especially amenable to cranial ultrasound since their skulls remain not yet ossified and their fontanelles open [125, 126]. Alterations in cerebral blood flow allow inferences to be made regarding brain pathology and raised intracranial pressure (ICP) [127, 128]. Estimation of flow velocities and calculation of pulsatility (PI) and resistance indexes (RI) are useful tools for non-invasive monitoring of ICP [129]. Age-dependent normal values of blood velocities in different vessels and indexes have been previously published [130]. Paediatric data are not yet sufficient to support its use and caution must be taken when interpreting results [131, 132].
  2. POCUS should be used to detect germinal matrix and intraventricular haemorrhage (IVH) in neonates—*strong agreement* (*quality of evidence A*). Intraventricular haemorrhage and parenchymal bleeding remain a frequent and serious complication in extremely preterm infants. POCUS is a useful clinical tool to detect IVH and parenchymal changes [133], and assesses the severity according to Papile’s classification [134, 135]. In environments where imaging resources are limited, brain POCUS should be used for the diagnosis of IVH which may aid in the redirection of care.
  3. POCUS is helpful to detect cerebral blood flow patterns suggesting the presence of cerebral circulatory arrest in children with fused skull bones—*agreement (quality of evidence C).* Transcranial doppler has been used to establish the presence of cerebral circulatory arrest [135, 136] by evaluation of the middle cerebral (MCA) and basilar artery. The following patterns are compatible with cerebral circulatory arrest [126, 136]: (a) oscillating waveform or sustained reversal of diastolic flow, (b) small systolic spikes and disappearance of all intra-cranial flow, (c) no flow in MCA, (d) reversal of diastolic flow in extracranial Internal Carotid Artery (ICA) and (e) mean velocity in MCA less than 10 cm/s for more than 30 min. However, interpretation of this assessment must be done with caution.
  4. POCUS is helpful to detect cerebral blood flow changes secondary to vasospasm in patients with traumatic brain injury and non-traumatic intracranial bleeding—*agreement (quality of evidence C).* Vasospasm of cerebral arteries results in increased flow velocities. The Lindegaard ratio (LR) is calculated by dividing mean velocity in MCA by mean velocity in ipsilateral extracranial ICA. As hyperaemia may also increase mean velocities, LR is useful to distinguish between hyperaemia and vasospasm (3:6 ratio is considered a sign of mild vasospasm, and > 6 a sign of severe vasospasm) [136].
  5. POCUS is helpful to detect changes in optic nerve sheath diameter (ONSD) indicative of raised ICP in children with fused skull bones—*agreement (quality of evidence B).* Measurement of the ONSD is suggestive of papilledema and increased ICP [137, 138]; however, data conflict on threshold measurements [137–140] and papilledema may persist despite normalisation of intracranial pressure. Therefore, it must be reminded that this technique may have relevant measurement errors because of the narrow margins that distinguish pathological from normal.
  6. POCUS is helpful to detect cerebral midline shift (MLS) in neonates and children—*agreement (quality of evidence C).* Measurement of the distance from both temporal bones to the centre of the third ventricle through the temporal acoustic window determines the presence of MLS [141, 142]. Minimal shifts (less than 5 mm) in adults have proven to identify abnormal conditions [143]. Yet, normative values for MLS identification in children have not been reported, and this may reduce the helpfulness of this application.
E. Recommendations for abdominal POCUS
  1. POCUS is helpful for the detection of free intra-abdominal fluid in neonates and children—*strong agreement (quality of evidence C).* Abdominal POCUS is widely used as a clinical tool for the management and diagnosis of patients with abdominal pathology [144–148]. Evaluation of free fluid is particularly useful when sudden clinical deterioration and hypotension occur and may provide insight into the ongoing pathophysiologic process [149, 150].
  2. POCUS may detect parenchymal changes of abdominal organs in neonates and children [although for a definite diagnosis a detailed assessment should be performed by a paediatric radiologist]—*agreement (quality of evidence D).* Basic ultrasound knowledge of abdominal organ anatomy is essential during POCUS examination. Furthermore, a detailed assessment should be performed by a paediatric radiologist if abnormal abdominal organ structures are detected by POCUS [151, 152].
  3. POCUS may detect obstructive uropathy in neonates and children [although for a definite diagnosis a detailed assessment should be performed by a paediatric radiologist]—*agreement (quality of evidence D)*. A renal ultrasound is useful to easily detect the presence of hydronephrosis even by novices [152–154]. Urinary retention can be evaluated by POCUS assessing postvoid residual volumes. In the case of anuria, a simple obstruction requiring a urinary catheter placement can be ruled out.
  4. POCUS may assess bowel peristalsis in neonates and children—*agreement (quality of evidence D).* Bowel POCUS may be used to assess peristalsis [155] but insufficient data yet exist correlating peristalsis with feeding tolerance. However, the presence of peristalsis has strong negative predictive value in adults for ileus and gut ischemia in adults [156].
  5. POCUS may recognise hypertrophic pyloric stenosis [although for a definitive diagnosis a detailed assessment should be performed by a paediatric radiologist]—*disagreement* (*quality of evidence D* )[157]
  6. POCUS may guide peritoneal drainage or aspiration of peritoneal fluid in neonates and children—*strong agreement (quality of evidence D).* A paracentesis may be required for either diagnostic or therapeutic purposes. POCUS should be used for pre-procedural planning, identification of epigastric vessels and real-time ultrasonographic guidance. In adult studies, the use of ultrasound has been shown to decrease both bleeding complications and the overall cost of care for patients undergoing in-hospital paracentesis [158].
  7. POCUS is helpful to detect signs of necrotizing enterocolitis (NEC) [although for a definitive diagnosis a detailed assessment should be performed by a paediatric radiologist or a person with specific advanced ultrasound training]—*agreement (quality of evidence C).* Ultrasound may be a useful adjunct detecting changes consistent with NEC even when radiographs are inconclusive [159, 160]. Furthermore, radiographs have poor sensitivity in the diagnosis of NEC [161, 162]. POCUS can provide prognostic value identifying free fluid, bowel wall thickness, *pneumatosis intestinalis*, portal venous gas and vascular perfusion [159, 163–166]. The International Neonatal Consortium’s NEC subgroup recently revisited the necrotizing enterocolitis (NEC) pathogenesis and new diagnostic criteria were proposed [167]. These were based on the so-called ‘two out of three’ model which includes *pneumatosis intestinalis* or portal venous gas by abdominal X-rays and/or POCUS. POCUS was included since various studies demonstrated that it outperformed conventional radiology to this end [159].

## Discussion

POCUS is increasingly being utilised in neonatal and paediatric critical care as a valuable adjunct to clinical examination. The use of POCUS by the clinician is different than a complete diagnostic study by the specialist and its role is dynamic—the same provider can perform and interpret the study, rapidly integrate this information within the current clinical setting, and then repeat the study to identify changes associated with the intervention. POCUS involves a focused assessment to answer a specific question, and it provides anatomical and/or physiological information to be integrated with clinical and laboratory data and make timely and accurate decisions possible. The statements on which panellists disagreed concerned two particular indications (endocarditis and hypertrophic pyloric stenosis), whose diagnosis should be done by a detailed ultrasonography in the hands of an expert paediatric cardiologist or radiologist. The role of POCUS (i.e., point-of-care ultrasound performed directly by neonatal/paediatric intensivists at the bedside) for these indications is not clear, while the role of ultrasound, *in general*, is indeed important.

**Fig. 2:**
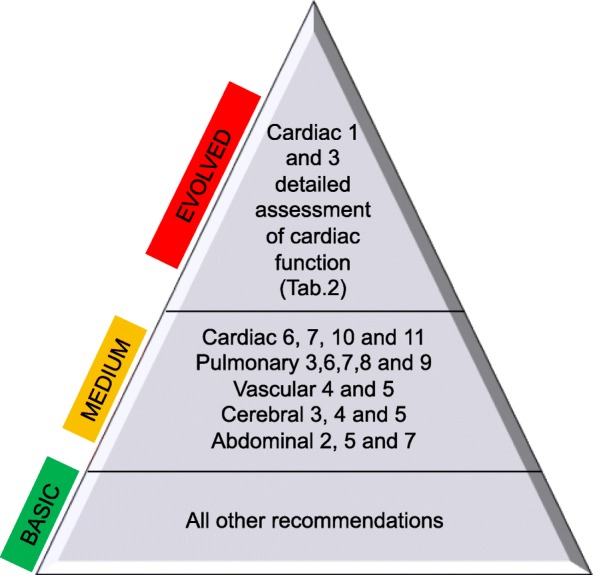
Estimated level of training required for the implementation of POCUS recommendations. Recommendations are listed according to their progressive number for each section

There remains a significant variation in clinical practice—in indications, training program and clinical governance. The application of POCUS in clinical practice is dependent on many factors including availability of ultrasound machines, providers, hospital setting, local patient population and specialty. Even within a unit, practice varies among clinicians and their expertise. We subdivided POCUS recommendations according to the estimated level of training required for their use (Fig. 2), and this may be helpful for their implementation.

Defining a scope of practice is fundamental for POCUS, and these ESPNIC guidelines have been developed for the targeted use of ultrasound in NICU and PICU by any neonatologist or paediatric intensivist. For most diagnostic (heart, lungs, brain and abdomen) and procedural (line placement and fluid drainage) POCUS applications, these guidelines can be used at all ages in children or neonates. However, while using cardiac POCUS in neonates, clinicians should be aware that undiagnosed critical congenital heart defects can present just during this period. They should acknowledge the limitations of skills, and it should not be used as a screening tool for diagnosing or excluding congenital heart defects. A patient with a suspected critical congenital heart defect should be quickly referred to a paediatric cardiologist, even if this implies out-of-hospital patient’s transportation. However, in cases of suspicion of a non-critical defect or for patients with low pre-test probability a more expectative management can be provided. Moreover, the use of pulse-oximetry screening for congenital heart defects is useful and should be integrated into the clinical decision-making process, as internationally recommended [168, 169].

These guidelines may help in standardising clinical practice across acute care settings and physicians. It is not meant to be prescriptive, but rather to outline the important features of structured POCUS applications in clinical practice. The use of these guidelines often will not have any cost implication for the equipment, as ultrasound machine is almost always available in most neonatal and paediatric intensive care units. ESPNIC will promote the diffusion of these guidelines by appropriate advertisement, links and presentation in the society congresses and training events.

These guidelines have strengths and limitations as any other. Strengths are represented by (1) the fairly subdivided multidisciplinary panellists’ group (including both neonatologists and paediatric intensivists, expert in different POCUS areas and coming from different geographical regions), and (2) the use of a standardised methodology. In several fields, these recommendations are based upon a few studies with moderate/low evidence or based upon experts’ opinions and this is a striking difference compared to similar guidelines in adult critical care. In fact, authors answered to following PICO questions: “Does point-of-care ultrasound for heart/lung/line placement/abdomen/brain provide any clinical advantage in neonatal or paediatric intensive care practice?” (supplementary methods). These questions were purposely quite general, as POCUS is not a therapeutic intervention, but rather a diagnostic and monitoring technique and we expected few randomised controlled trials or high-quality diagnostic accuracy studies about POCUS specifically aiming to improve any particular paediatric/neonatal outcome. Moreover, some of the POCUS applications may not be immediately used in all intensive care units as in ours, because of the specific expertise required in some particular contexts (such as the extremely preterm neonates or non-sedated and non-collaborative patients). However, the existence of formal guidelines may help to improve the local expertise and foster new studies to expand knowledge in this field and refine future recommendations. This is extremely important as further studies are warranted to demonstrate that POCUS actually improves patients’ management and outcome, similar to what has been demonstrated in adult critical care [1–7, 55]. Another concern is that the clinicians involved are all enthusiastic users of POCUS and this may have theoretically contributed to have ‘too positive’ recommendations. The ESPNIC POCUS Working Group recognises that there are emerging indications for POCUS outside of those listed here and needs for future research steps. Publication of new literature may require future revision and ESPNIC POCUS Working Group is willing to update guidelines accordingly every 3 years.

These guidelines may provide a substrate to develop a POCUS curriculum and structured training programs, which are urgently needed for quality assurance. This guideline paper is not a training statement: requirements and methods of POCUS training should be a sensible follow-up article to this one.

## Conclusions

Despite the lack of published evidence-based guidelines specifically for its use in the neonatal and paediatric intensive care units, POCUS is increasingly being used. ESPNIC POCUS guidelines provide the substrate to optimise POCUS use in neonatal and paediatric critical care and guide future research steps according to currently unmet needs. Guidelines may help in making the clinical practice standardised and clinical governance robust.

## Supplementary information

**Additional file 1.** Online supplementary material.

## Data Availability

Data sharing is not applicable to this article as no datasets were generated or analysed during the current study.

## Abbeviations

A4C: Apical 4-chamber
ARDS: Acute respiratory distress syndrome
CPU: Clinician performed ultrasound
EF: Ejection fraction
ESPNIC: European Society of Paediatric and Neonatal Intensive Care
FS: Fraction shortening
ICP: Intracranial pressure
IJV: Internal jugular vein
IVC: Inferior vena-cava
IVH: Intraventricular haemorrhage
LR: Lindegaard ratio
LUS: Lung ultrasound
LVOT: Left ventricular outflow tract
MAS: Meconium aspiration syndrome
MCA: Middle cerebral artery
MLS: Cerebral midline shift
NEC: Necrotizing enterocolitis
NICU: Neonatal intensive care unit
NPE: Neonatologist performed echocardiography
ONSD: Optic nerve sheath diameter
PASP: Pulmonary artery systolic pressure
PI: Pulsatility index
PICC: Peripherally inserted central catheters
PICU: Pediatric intensive care unit
PLAX: Parasternal long
POCUS: Point of care ultrasound
PSAX: Short axis
RDS: Respiratory distress syndrome
RI: Resistance index
TTN: Transient tachypnoea of the neonate
VTIs: Variation of velocity-time integrals
