# Supplementary material for: International evidence-based guidelines on Point of Care Ultrasound (POCUS) for critically ill neonates and children issued by the POCUS Working Group of the European Society of Paediatric and Neonatal Intensive Care (ESPNIC)
Source: Crit Care. 2020 Feb 24;24:65. doi: 10.1186/s13054-020-2787-9 (PMC7041196; doi:10.1186/s13054-020-2787-9)
Supplement: Supplementary file 1 — Additional file 1. Online supplementary material. [file 13054_2020_2787_MOESM1_ESM.docx]

**Additional file 1: Online Supplementary material**

**MATERIAL AND METHODS**

The whole project has been divided in two phases: 1) the assessment of the level of quality of evidence and 2) the development of the recommendations. The conversion of evidence into recommendations depends on the evaluation of possible benefits and costs (and their ratio), but also possible logistical and technical difficulties, training issues, differences across patients’ age and settings and certainty about similarity in preferences.

As for the first phase (assessment of the level of quality of evidence), an electronic literature search in PubMed was incepted (27 Jan 2019) for each area of point-of-care ultrasound (POCUS) application using the keywords listed below:

***Heart*** *("echocardiography"[MeSH Terms] OR "echocardiography"[All Fields]) AND ("infant, newborn"[MeSH Terms] OR ("infant"[All Fields] AND "newborn"[All Fields]) OR "newborn infant"[All Fields] OR "neonate"[All Fields]) and ("echocardiography"[MeSH Terms] OR "echocardiography"[All Fields]) AND ("child"[MeSH Terms] OR "child"[All Fields] OR "children"[All Fields]);* ***Lung*** *("lung"[MeSH Terms] OR "lung"[All Fields]) AND ("diagnostic imaging"[Subheading] OR ("diagnostic"[All Fields] AND "imaging"[All Fields]) OR "diagnostic imaging"[All Fields] OR "ultrasound"[All Fields] OR "ultrasonography"[MeSH Terms] OR "ultrasonography"[All Fields] OR "ultrasound"[All Fields] OR "ultrasonics"[MeSH Terms] OR "ultrasonics"[All Fields]) AND ("infant, newborn"[MeSH Terms] OR ("infant"[All Fields] AND "newborn"[All Fields]) OR "newborn infant"[All Fields] OR "neonate"[All Fields]) and ("lung"[MeSH Terms] OR "lung"[All Fields]) AND ("diagnostic imaging"[Subheading] OR ("diagnostic"[All Fields] AND "imaging"[All Fields]) OR "diagnostic imaging"[All Fields] OR "ultrasound"[All Fields] OR "ultrasonography"[MeSH Terms] OR "ultrasonography"[All Fields] OR "ultrasound"[All Fields] OR "ultrasonics"[MeSH Terms] OR "ultrasonics"[All Fields]) AND ("child"[MeSH Terms] OR "child"[All Fields] OR "children"[All Fields]);* ***Line placement*** *("blood vessels"[MeSH Terms] OR ("blood"[All Fields] AND "vessels"[All Fields]) OR "blood vessels"[All Fields] OR "vascular"[All Fields]) AND access[All Fields] AND ("diagnostic imaging"[Subheading] OR ("diagnostic"[All Fields] AND "imaging"[All Fields]) OR "diagnostic imaging"[All Fields] OR "ultrasound"[All Fields] OR "ultrasonography"[MeSH Terms] OR "ultrasonography"[All Fields] OR "ultrasound"[All Fields] OR "ultrasonics"[MeSH Terms] OR "ultrasonics"[All Fields]) AND ("infant, newborn"[MeSH Terms] OR ("infant"[All Fields] AND "newborn"[All Fields]) OR "newborn infant"[All Fields] OR "neonate"[All Fields]) and ("blood vessels"[MeSH Terms] OR ("blood"[All Fields] AND "vessels"[All Fields]) OR "blood vessels"[All Fields] OR "vascular"[All Fields]) AND access[All Fields] AND ("diagnostic imaging"[Subheading] OR ("diagnostic"[All Fields] AND "imaging"[All Fields]) OR "diagnostic imaging"[All Fields] OR "ultrasound"[All Fields] OR "ultrasonography"[MeSH Terms] OR "ultrasonography"[All Fields] OR "ultrasound"[All Fields] OR "ultrasonics"[MeSH Terms] OR "ultrasonics"[All Fields]) AND ("child"[MeSH Terms] OR "child"[All Fields] OR "children"[All Fields]);* ***Abdomen****("abdominal cavity"[MeSH Terms] OR ("abdominal"[All Fields] AND "cavity"[All Fields]) OR "abdominal cavity"[All Fields] OR "abdomen"[All Fields] OR "abdomen"[MeSH Terms]) AND ("diagnostic imaging"[Subheading] OR ("diagnostic"[All Fields] AND "imaging"[All Fields]) OR "diagnostic imaging"[All Fields] OR "ultrasound"[All Fields] OR "ultrasonography"[MeSH Terms] OR "ultrasonography"[All Fields] OR "ultrasound"[All Fields] OR "ultrasonics"[MeSH Terms] OR "ultrasonics"[All Fields]) AND ("infant, newborn"[MeSH Terms] OR ("infant"[All Fields] AND "newborn"[All Fields]) OR "newborn infant"[All Fields] OR "neonate"[All Fields]) and ("abdominal cavity"[MeSH Terms] OR ("abdominal"[All Fields] AND "cavity"[All Fields]) OR "abdominal cavity"[All Fields] OR "abdomen"[All Fields] OR "abdomen"[MeSH Terms]) AND ("diagnostic imaging"[Subheading] OR ("diagnostic"[All Fields] AND "imaging"[All Fields]) OR "diagnostic imaging"[All Fields] OR "ultrasound"[All Fields] OR "ultrasonography"[MeSH Terms] OR "ultrasonography"[All Fields] OR "ultrasound"[All Fields] OR "ultrasonics"[MeSH Terms] OR "ultrasonics"[All Fields]) AND ("child"[MeSH Terms] OR "child"[All Fields] OR "children"[All Fields]);* ***Brain*** *("brain"[MeSH Terms] OR "brain"[All Fields]) AND ("diagnostic imaging"[Subheading] OR ("diagnostic"[All Fields] AND "imaging"[All Fields]) OR "diagnostic imaging"[All Fields] OR "ultrasound"[All Fields] OR "ultrasonography"[MeSH Terms] OR "ultrasonography"[All Fields] OR "ultrasound"[All Fields] OR "ultrasonics"[MeSH Terms] OR "ultrasonics"[All Fields]) AND ("infant, newborn"[MeSH Terms] OR ("infant"[All Fields] AND "newborn"[All Fields]) OR "newborn infant"[All Fields] OR "neonate"[All Fields]) and ("brain"[MeSH Terms] OR "brain"[All Fields]) AND ("diagnostic imaging"[Subheading] OR ("diagnostic"[All Fields] AND "imaging"[All Fields]) OR "diagnostic imaging"[All Fields] OR "ultrasound"[All Fields] OR "ultrasonography"[MeSH Terms] OR "ultrasonography"[All Fields] OR "ultrasound"[All Fields] OR "ultrasonics"[MeSH Terms] OR "ultrasonics"[All Fields]) AND ("child"[MeSH Terms] OR "child"[All Fields] OR "children"[All Fields])*

Clinical studies respecting the following criteria were considered: 1) patients’ age under 18 years, 2) use of POCUS by neonatal and paediatric intensivist only [i.e.: studies not performed in NICUs and PICUs were excluded]. If for a specific area, no studies were available on patients under 18 years of age, the search was repeated looking for articles on adult patients reporting the use of POCUS by adult critical care physicians in intensive care units. Case series/reports, animal or translational studies and grey literature were not considered. No language or year restrictions were applied. Authors searched for articles also in their personal archives and through the reference list of relevant articles identified through the searches above. These searches were derived by the following PICO questions “*Does point-of-care ultrasound for heart/lung/line placement/abdomen/brain provide any clinical advantage in neonatal or paediatric intensive care practice?*”. These questions were purposely quite general as point-of-care lung ultrasound is not a therapeutic intervention, but rather a diagnostic and monitoring technique and we expected a lack of randomised controlled trials of POCUS specifically aiming to improve a particular outcome. For these reasons, it was considered unlikely to find POCUS to have a particular effect on specific outcomes. Panellists were divided in subgroups based upon their expertise [cardiac, lung, brain, line placement and abdomen] and a subgroup for each of the five areas was created. Subgroups screened the references of their field by reading the abstract and, where needed, the full text.

As for the second phase (development of recommendations), every subgroup critically analysed the literature in their area through serial videoconferences. During these meetings, the literature was classified per level of quality of evidence and grade of recommendations according the GRADE system [E1] as previously done for the international guidelines on point-of-care lung ultrasound in adult patients. [E2] When only papers on adults were available, they were considered but their level of quality of evidence was lowered. After literature analysis, every subgroup drafted their recommendations by internal discussion and subgroups recommendations were discussed collegially by all panellists in Nov 2018, during a dedicated in-person meeting, moderated by the one of the project coordinator (YS) within the annual ESPNIC meeting. All the discussions were organised through the ESPNIC secretariat and followed a Quaker-based technique which included open discussion, active listening, sharing of information and questions with no restriction. (E3)

The discussion resulted in a final set of recommendations, subjected to electronic, anonymous voting (during January 2019) using a modified Research and Development/ University of California, Los Angeles (RAND/UCLA) appropriateness method scale, (E4) as previously done for ESPNIC mechanical ventilation guidelines. (E5) The voting was organised by the ESPNIC secretariat using ESPNIC online platform. Recommendations were scored from 1 (complete disagreement) to 9 (complete agreement). Median score (95% confidence interval) was calculated after eliminating one lowest and highest value. Recommendations were labelled “strong agreement” if they had a median score comprised between 7 and 9 and no score <7. Recommendations without “strong agreement” were re-discussed in a videoconference with all panellists, rephrased and subjected to a second voting round (during February 2016): they were finally labelled “agreement” (median score comprised between 7 and 9 and no score <4) or “disagreement” (median score comprised between 1 and 3). At the second voting round, no recommendation fulfilled the criteria for strong agreement and there were no recommendations with low median score (that is, comprised between 4 and 6).

**E-REFERENCES**

**E1**. Volpicelli G, Elbarbary M, Blaivas M, Lichtenstein DA, Mathis G, Kirkpatrick AW, Melniker L, Gargani L, Noble VE, Via G, Dean A, Tsung JW, Soldati G, Copetti R, Bouhemad B, Reissig A, Agricola E, Rouby JJ, Arbelot C, Liteplo A, Sargsyan A, Silva F, Hoppmann R, Breitkreutz R, Seibel A, Neri L, Storti E, Petrovic T; International Liaison Committee on Lung Ultrasound (ILC-LUS) for International Consensus Conference on Lung Ultrasound (ICC-LUS) (2012) [International evidence-based recommendations for point-of-care lung ultrasound.](https://www.ncbi.nlm.nih.gov/pubmed/22392031) Intensive Care Med 38:577-91. doi: 10.1007/s00134-012-2513-4.

**E2**. Quaker reference: Quaker Foundations of Leadership. A comparison of Quaker-based consensus and Robert’s rules of order. Richmond (VI), USA: Earlham College, 1999.

**E3**. Atkins D, Best D, Briss PA, Eccles M, Falck-Ytter Y, Flottorp S, Guyatt GH,

Harbour RT, Haugh MC, Henry D, Hill S, Jaeschke R, Leng G, Liberati A,

Magrini N, Mason J, Middleton P, Mrukowicz J, O’Connell D, Oxman AD,

Phillips B, Schunemann HJ, Edejer T, Varonen H, Vist GE, Williams JW Jr,

Zaza S, Group GW (2004) Grading quality of evidence and strength of

recommendations. BMJ 328:1490.

**E4**. Fitch K, Bernstein SJ, Aguilar MD, Burnand B, LaCalle JR, Lazaro P, van het

Loo M, McDonell J, Vader JP, Kahan JP (2001) The RAND/UCLA appropriateness

method user’s manual. RAND, Santa Monica (CA), USA.

**E5**. Kneyber MCJ, de Luca D, Calderini E, Jarreau PH, Javouhey E, Lopez-Herce J, Hammer J, Macrae D, Markhorst DG, Medina A, Pons-Odena M, Racca F, Wolf G, Biban P, Brierley J, Rimensberger PC; section Respiratory Failure of the European Society for Paediatric and Neonatal Intensive Care (2017) [Recommendations for mechanical ventilation of critically ill children from the Paediatric Mechanical Ventilation Consensus Conference (PEMVECC).](https://www.ncbi.nlm.nih.gov/pubmed/28936698) Intensive Care Med 43:1764-1780.
